# 

**Published:** 1876-02

**Authors:** 


### CONTENTS:

### ORIGINAL COMMUNICATIONS.

ARTICLE I.—Virginia State Dental Association—Sixth Annual Session........... 433

ARTICLE II.—On the Preservation of the Deciduous Teeth. By N. M. Burkholder, D. D. S........ 445

ARTICLE III—Are Irregularities of the Teeth ever caused by Tongue and Thumb Sucking? By Jas. F. Thompson, D. D. S., M. D............... 455

ARTICLE IV.—American Dental Association. By J. H. McQuillen, M. D., D. D. S. 458

ARTICLE V.—American Academy of Dental Science. By Geo. T. Moffatt, M. D.. 460

### SELECTED ARTICLES.

ARTICLE VI—Important Decision in the Celluloid Case.................. 461

ARTICLE VII—Osteo-Sarcoma of Lower Jaw. By C. S. Briggs, M. D.............. 464

ARTICLE VIII—Treatment of Exposed Pulps. By Dr. D. D. Peabody........... 467

### EDITORIAL, ETC.

Alumni Meeting.................. 470
Southern Dental Association.................. 471
Ether Spray as an Anæsthetie.................. 471

### MONTHLY SUMMARY.

Treatment of Neuralgia.................. 473

Treatment of Facial Neuralgia.................. 474

Antidote to Carbolic Acid.................. 474

Plastic Operation for Lower Lip.................. 475

Borate of Soda Gargles.................. 476

Quinine Gargle in Sore Throat.................. 476

Glycosuria.................. 477

New Remedy for Burns.................. 477

Making a New Nose out of a Finger.................. 477

Application to Burns.................. 478

Mode of Administering Ether.................. 478

Chloral for Ulcers.................. 479

Antiseptic Properties of Salicylic and Benzoic Acids.................. 480

Glass Cement.................. 480

